# Recombinant growth hormone improves growth and adult height in patients with maternal inactivating *GNAS* mutations

**DOI:** 10.1093/ejendo/lvad085

**Published:** 2023-07-20

**Authors:** Diana-Alexandra Ertl, Guiomar Perez de Nanclares, Harald Jüppner, Patrick Hanna, Angela Pagnano, Arrate Pereda, Anya Rothenbuhler, Giulia Del Sindaco, Pilar Ruiz-Cuevas, Christelle Audrain, Arancha Escribano, Jugurtha Berkenou, Andreas Gleiss, Giovanna Mantovani, Agnès Linglart

**Affiliations:** 1 Department of Endocrinology and Diabetology for Children and Department of Adolescent Medicine, AP-HP, Bicetre Paris-Saclay University Hospital, 94270 Le Kremlin-Bicetre, France; 2 Reference Center for Rare Disorders of the Calcium and Phosphate Metabolism, AP-HP, Filière OSCAR and Platform of expertise for rare diseases Paris-Saclay, Bicêtre Paris-Saclay Hospital, 94270 Le Kremlin-Bicêtre, France; 3 Department of Paediatrics and Adolescent Medicine, Medical University of Vienna, 1090 Vienna, Austria; 4 Reference Center for Rare Bone and Growth Disorders, Vienna Bone and Growth Center (ERN-BOND), 1090 Vienna, Austria; 5 Molecular (Epi) Genetics Laboratory, Bioaraba Health Research Institute, Araba University Hospital, 01009 Vitoria-Gasteiz, Spain; 6 Department of Medicine, Endocrine Unit, Massachusetts General Hospital and Harvard Medical School, Boston, MA 02114, United States; 7 Endocrinology Unit, Fondazione IRCCS Ca’ Granda Ospedale Maggiore Policlinico, 20122 Milan, Italy; 8 Department of Clinical Sciences and Community Health, University of Milan, 20122 Milan, Italy; 9 Department of Pediatric Endocrinology, Josep Trueta University Hospital, 17007 Girona, Spain; 10 Department of Pediatric Endocrinology, University Hospital Virgen de la Arrixaca, 30120 El Palmar, Murcia, Spain; 11 Center for Medical Data Science, Medical University of Vienna, 1090 Vienna, Austria; 12 INSERM, Physiologie et physiopathologie endocrinienne, Université Paris Saclay, 94276 Paris, France

**Keywords:** pseudohypoparathyroidism, recombinant human growth hormone, pediatric, short stature, iPPSD

## Abstract

**Background:**

Maternal inactivating *GNAS* mutations lead to pseudohypoparathyroidism 1A (PHP1A), newly classified as inactivating parathyroid hormone (PTH)/PTHrP-signaling disorder type 2 of maternal inheritance (iPPSD2). Patients present with resistance to PTH and other hormones, subcutaneous ossifications, brachydactyly, short stature, and early-onset obesity. They can be born small for gestational age (SGA) and may present with growth hormone (GH) deficiency. The use of recombinant human GH (rhGH) therapy has been sporadically reported, yet we lack data on the long-term efficacy and safety of rhGH, as well as on adult height.

**Objective:**

Our multicenter, retrospective, observational study describes growth in patients treated with rhGH in comparison with untreated iPPSD2/PHP1A controls.

**Methods:**

We included 190 patients, of whom 26 received rhGH. Height, weight, body mass index at various time points, and adult height were documented. We analyzed the effect of rhGH on adult height by using linear mixed models.

**Results:**

Adult height was available for 11/26 rhGH-treated individuals and for 69/164 controls. Patients treated with rhGH showed a gain in height of 0.7 standard deviation scores (SDS) after 1 year (CI +0.5 to +0.8, *P* < .001) and of 1.5 SDS after 3 years (CI +1.0 to +2.0, *P* < .001). Additionally, there was a clear beneficial impact of rhGH on adult height when compared with untreated controls, with a difference of 1.9 SDS (CI +1.1 to +2.7, *P* < .001). Body mass index SDS did not vary significantly upon rhGH therapy.

**Conclusion:**

Recombinant human growth hormone treatment of iPPSD2/PHP1A patients with short stature improves growth and adult height. More studies are needed to confirm long-term efficacy and safety.

## Introduction

Pseudohypoparathyroidism (PHP) was initially defined as a metabolic disorder characterized by resistance to parathyroid hormone (PTH), resulting in hypocalcemia and hyperphosphatemia, associated with specific features of Albright hereditary osteodystrophy, such as stocky build, brachydactyly, and short stature.^1^ Additional features, such as subcutaneous ossifications, early-onset obesity, and resistance to other hormones, were later described in PHP patients. Pseudohypoparathyroidism 1A (PHP1A) is caused by inactivating variants involving the maternal allele of the *GNAS* gene encoding Gsα, the alpha-subunit of the stimulatory G protein.^2,3^ Pseudohypoparathyroidism 1A belongs to a large group of rare diseases that share common clinical features such as brachydactyly and end-organ resistance to PTH because of impaired signaling downstream of the PTH1R and other G protein-coupled receptors (GPCRs). The molecular defects causing these disorders all affect the PTH/PTHrP-signaling pathway, which involves Gsα, cAMP, and protein kinase A (PKA), and is necessary for the actions of other hormones, such as thyroid-stimulating hormone (TSH), growth hormone–releasing hormone (GHRH), gonadotropins, and calcitonin.^2,4-11^ Because of the clinical and molecular overlap of these diseases, a novel disease classification has been established under the term “inactivating PTH/PTHrP-signaling disorder” (iPPSD) based on the common impaired signaling of the PTH/PTHrP receptor; the advantages and limitations, including the impaired signaling downstream of other GPCRs, of this classification have been described.^12^ iPPSD2mat refers to patients bearing loss-of-function mutations in the *GNAS* gene,^11,12^ and we will refer to this patient group as iPPSD2/PHP1A.

A progressive decline of height velocity and an adult height below −2 standard deviation scores (SDS) in individuals with iPPSD2/PHP1A have been previously reported, mostly as published series of cases.^2,9,11,13,14^ Few observational studies have been conducted using larger cohorts.^15-17^ Our research group published in 2018 the progression of height and body mass index (BMI) in the largest cohort investigated up to that point, showing that more than half of iPPSD2/PHP1A individuals present with short stature and early-onset obesity.^14^ Obesity has also been reported as a feature of iPPSD2/PHP1A by others.^5,18,19^

PTHrP and PTH-receptor 1 (PTH1R) play a major role in endochondral ossification;^20,21^ thus, it would be expected that a dysfunctional signaling pathway may result in an acceleration of chondrocyte differentiation, advanced skeletal maturation, and short stature. The main factors suspected to be responsible for the height deficit in iPPSD2/PHP1A include a history of small for gestational age (SGA), often reported in iPPSD2, growth hormone deficiency (GHD) due to GHRH resistance, hypothyroidism due to TSH resistance, and advanced skeletal maturation due to PTHrP resistance, as well as obesity.^2,9^ Recombinant human GH (rhGH) therapy is indicated in short children born SGA or diagnosed with GHD; therefore, a subset of patients with iPPSD2/PHP1A may have been treated with rhGH, yet data on efficacy, adult height, and safety are lacking. The series of cases published so far, reporting growth during rhGH treatment, were either conducted for only a short period of time^22-24^ or lacked a control population.^25^

Therefore, we considered of great importance the analysis of growth progression and adult height in our large cohort of individuals with iPPSD2/PHP1A treated with rhGH and compared them with untreated iPPSD2/PHP1A controls.

## Methods

### Study design and population

We conducted a multicenter observational study on a cohort of individuals clinically, biochemically, and genetically diagnosed with iPPSD2/PHP1A from France, Italy, and Spain, previously investigated by our group.^14^ Briefly, patients were included only if they are carriers of a loss-of-function mutation on the maternal allele of the *GNAS* gene, thus confirming the diagnosis of iPPSD2mat/PHP1A. Within this patient population, we identified individuals who were treated with rhGH, but their growth during this therapy was not included in the report by Hanna et al.^14^ This cohort of rhGH-treated patients was extended by adding new cases from the participating countries, which were then documented prospectively. To analyze the potential effect of this therapy on growth and adult height, we compared these participants to untreated iPPSD2/PHP1A individuals (hereafter designated as controls) from the historic cohort.^14^

In accordance with the Jardé law in France, as the study was approved by the French Data Protection Authority (CNIL), the need for written consent was waived. Patients and/or their parents/legal guardians were informed verbally of the objectives and procedures of the study, and their consent was obtained. They have the right to refuse to participate or to withdraw at any time by writing to http://recherche.aphp.fr/eds/droit-opposition. All other centers were approved by local ethics committees for the collection of data on the patients, as mentioned in our previous work.^14^

### Auxological and biochemical measurements

**Figure 1.**
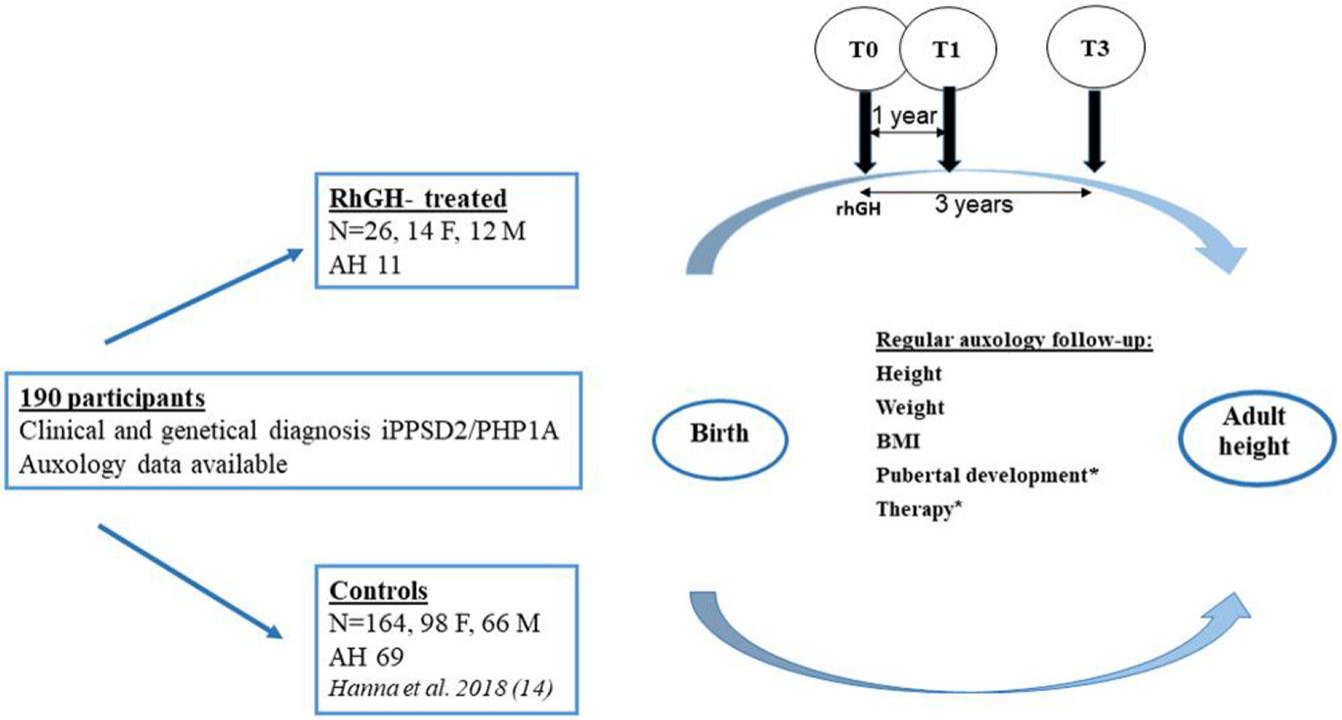
Study design. *Only for individuals from the rhGH-treated group. F, female; M, male; T0, baseline; T1, first year of therapy; T3, 3 years of therapy; AH, adult height; rhGH, recombinant human growth hormone; BMI, body mass index.

Auxological data (height [cm], weight [kg], BMI [kg/m^2^]), starting from birth, were gathered from medical charts. We used the already available measurements from the historical cohort^14^ for the controls and for the individuals in the rhGH group before rhGH therapy was started, at the following time points: birth, 0.5, 1, 1.5, 2, 3, 6, 8, 10, 12, 14, 16, and 18 years. Final adult height was defined as height at the age of 18. The same time points were used for the newly added participants in both groups. For the rhGH-treated iPPSD2/PHP1A participants, additional data (eg, serum insulin-like growth factor I [IGF-I] [ng/mL], rhGH dose [mg/kg/day], and pubertal status) were collected during rhGH treatment. Additionally, height measurements 1 year before the start of rhGH were included, in order to assess pretreatment growth. These auxological parameters, as well as BMI, were analyzed as SDS, using WHO reference data.^26^ Differences in height and BMI SDS (ΔH, ΔBMI) in comparison with baseline (T0) were calculated for the rhGH-treated participants after the first (T1) and third year (T3) of therapy. A schematic representation of the study population and design is shown in Figure 1.

Parental height and genetic target height were documented for all patients, but these data were not included in the analysis due to the high proportion of familial cases in the study population, which would have biased the interpretation.

We also planned to gather information on bone age, but the severity of the skeletal phenotype on some radiographs hindered accurate analysis of bone maturation, so we decided not to analyze this parameter. Routine biochemical assessment was performed according to follow-up guidelines,^13^ for example, thyroid function, IGF-I, calcium and phosphate metabolism. All individuals presented no other conditions that might additionally contribute to short stature.

### Growth-promoting treatment

The medical indication for rhGH therapy in most of the treated population was either SGA or GHD, in accordance with the existing guidelines.^27-29^ The latter was proven by means of basal IGF-I measurements followed by GH stimulation tests, in agreement with the existing guidelines.^27,28^ In 3 patients, the indication was made after careful expert discussion because of advanced bone maturation and poor adult height prognosis.

All rhGH-treated participants received the subcutaneous injection 6 days per week. The dose of rhGH was regularly adjusted based on height velocity, weight, and serum IGF-I concentration. The target was an IGF-I concentration in the normal range for age, gender, and pubertal stage.

A subset of children with early onset of puberty, advanced bone age, and unfavorable predicted adult height (<−2 SDS) was additionally treated with a gonadotropin-releasing hormone (GnRH) analog. The decision was made collectively by the pediatric endocrinology team and the families during information meetings. Children received triptorelin or leuprorelin, mainly using the 3-month sustained-release formulation, at a dose of 11.25 mg.^30^

### Statistical analysis

Continuous variables are described using mean, SD and range (minimum and maximum) in the case of an approximate normal distribution, and using median, quartiles, and range otherwise. To analyze the effect of rhGH therapy after 1 and 3 years of treatment, the normed 1-year difference of height SDS or BMI SDS, respectively (ie, first year of treatment for patients under treatment; approximately 1-year periods otherwise), was compared between the conditions “under rhGH therapy” and “never or not yet under therapy” (including control patients as well as rhGH-treated patients before treatment started), using a linear mixed model that includes an adjustment for age and a random patient factor (to account for repeated measurements within patients). In order to investigate if the treated individuals and the controls were different in terms of height before the rhGH treatment (implying a potential preselection of shorter children as candidates for rhGH therapy), all participants from the rhGH-treated group without treatment before the age of 8 years were selected, as were controls with available height measurements before the same age. The potential influence of rhGH therapy on adult height was then investigated using a linear regression model adjusted for the mean height SDS across measurements up to the age of 8 for the same individuals, selected as previously described.

The mean differences for both height and BMI SDS were estimated with 95% confidence intervals. *P*-values below .05 were considered to indicate statistical significance.

All analyses were based on the available measurements. In the rhGH-treated group, unavailable measurements at T3 and of adult height were due to the fact that some patients were still under treatment at the end of the observation period. The observed cases may be viewed as representative of all treated patients. In the control group, data are mixed cross-sectional and longitudinal from a well-defined cohort; however, we cannot exclude selection bias regarding the number and timing of measurements in the control group.

The patients who received both rhGH and GnRH analogs are described in this report; due to the small sample size, we could not perform statistical analysis on this subgroup.

## Results

### Characteristics of the study population

Our study cohort comprised 190 participants diagnosed with iPPSD2/PHP1A. As already mentioned, the clinical and biochemical diagnosis was confirmed by genetic analysis, thus excluding the possibility of misdiagnosis due to phenotypic overlap. We identified within this cohort 26 participants who were treated with rhGH for at least 1 year (rhGH-treated group), that is, 6 from Spain and 20 from France. Some of them were still undergoing treatment at the end of the observation period. The rest of the individuals never received rhGH and they were used in the analysis as controls (Figure 1).

In the *rhGH-treated group*, 14 out of 26 individuals are females. At the time of the analysis, all patients had been treated with rhGH for at least 1 year, 19 for at least 2 years, and 14 for at least 3 years. Beside the participants who reached adult height, the remaining patients are still under rhGH treatment. Six of the 24 participants (2 cases of missing data) were born SGA. In this cohort, we also encountered 6 cases of premature birth. Median birth length SDS was −1.2 (min/max: −5.2/0.5), while median birth BMI SDS was 0 (min/max: −8.1/1.9; Table 1). The median number of height measurements per participant available for this group during the observation period was 11 (min/max: 6/15).

All individuals in this group had elevated PTH concentrations at diagnosis, while 23 of them also presented with TSH resistance. All participants were adequately treated for their PTH and TSH resistances during the observation period, as documented in their clinical and biochemical reports. Age was between 2.3 and 13.6 years at the initiation of rhGH treatment (T0), with a median of 9 years. Four participants had already entered puberty at the start of rhGH (2 patients in Tanner stage II, 1 patient in stage III, and 1 patient in stage IV). Eleven participants received rhGH for at least 3 years and reached adult height. They were treated for a median of 5.2 years (min/max: 2.5/7.0 years). Recombinant human growth hormone doses (μg/kg/day) during the observation period remained constant, with a median value of 57 μg/kg/day at T0 and 61 μg/kg/day at T3. Insulin-like growth factor I concentrations, documented in ng/mL, increased after the start of rhGH, but remained in the normal range for Tanner stage in all participants with available measurements. After 1 year of therapy, the range (minimum-maximum) of available IGF-I concentrations was as follows: Tanner I (*n* = 12): 203-431 ng/mL (n.r. 126-496), Tanner II (*n* = 2): 353-466 ng/mL (n.r. 198-551), and Tanner III (*n* = 1): 164 ng/mL (n.r. 238-672). After 3 years of rhGH therapy: Tanner I (*n* = 3): 67-296 ng/mL, Tanner II (*n* = 2): 292-595 ng/mL, and Tanner III (*n* = 1): 328 ng/mL. During the period of observation, none of the patients reported tumors, scoliosis, hip epiphysiodesis intracranial hypertension, or papilledema.

When rhGH was started (T0), median/lower and upper quartile for height SDS were −1.2/−2.0 to −0.7 (min/max: −3.8 to 1 SDS) and 1.9/0.9 to 2.6 SDS (min/max: −2.5 to 4 SDS) for BMI, respectively. Nine patients presented a BMI greater than +2 SDS at baseline. Changes in height and BMI SDS during rhGH treatment are shown in Table 2. The gain in height SDS was 0.6 ± 0.5 after 1 year and 1.3 ± 1.0 SDS over the first 3 years of rhGH therapy.

Body mass index SDS did not change on average under this treatment.

Eleven individuals (7 females) from this group reached adult height (Table 1), with a median of −0.8 SDS (min/max: −2.5/0.4); only 1 patient presented with an adult height <−2 SDS.

The *control group* comprised 164 individuals, 98 of whom were females. According to the available measurements, the incidence of SGA individuals was comparable with that of the rhGH-treated group (26/99). Median birth length SDS was higher in the controls (−0.9 [min/max: −6.3/3.7]), than in the rhGH group (−1.2 [min/max: −5.2/0.5]), while median birth BMI SDS was comparable (0.1 [min/max: −5.6/3.5] and 0 [min/max: −8.1/1.9], respectively; Table 1). Prepubertal height measurements and adult height data were available for 22 and 69 controls; respectively. Body mass index SDS at the age of 18 was available for 52 of them. At adult height, the rhGH-treated patients were taller than the control patients, with a median height of −2.4 and −0.8 SDS (Table 1), respectively. In addition, 41/69 iPPSD2/PHP1A controls have an adult height <−2 SDS.

In this group, the median BMI at the age of 18 (*n* = 52) was 1.5 SDS (min/max: −1.0 to 3.8 SDS, lower upper quartile: 1 to 2.5 SDS); 37 of these iPPSD2/PHP1A controls presented with values in the upper normal range or above.

### Efficacy of rhGH therapy: Comparison of growth between rhGH-treated patients and controls

We looked at both height and BMI SDS during the first 3 years of rhGH therapy and compared the rhGH-treated individuals to controls and treated individuals before the start of treatment (never treated or not yet treated).

#### Efficacy of 1 year of rhGH therapy

**Figure 2.**
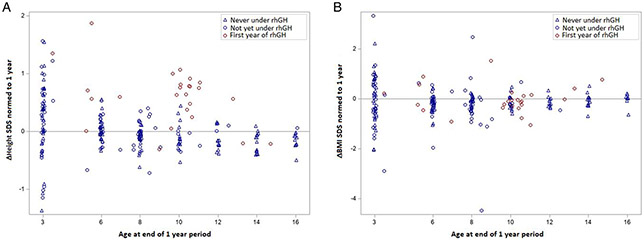
Therapeutic effect of rhGH therapy after 1 year: (A) change in height; (B) change in BMI. Gray line indicates 0 SDS, representing no change in height. rhGH, recombinant human growth hormone; BMI, body mass index; SDS, standard deviation score.

The mean increase in height SDS change over a treatment period of 1 year was 0.7 SDS (CI 0.5-0.8, *P* < .001) when compared with controls and patients not yet treated. Figure 2A shows the change in height SDS (ΔHeight) over the period of 1 year, for both controls and rhGH-treated participants, for all available measurements at the ages of interest. The mixed-model approach allows each treated participant to serve as control before treatment. The changes in height SDS over the span of 1 year (ΔHeight) for rhGH-treated participants (red circles) are clearly greater than the values recorded for the untreated participants (blue circles and blue triangles).

The same model was used for BMI SDS, but the estimated difference of 0.1 SDS (CI −0.1 to 0.4) was not statistically significant (*P* = .280; Figure 2B).

#### Efficacy of 3 years of rhGH therapy

**Figure 3.**
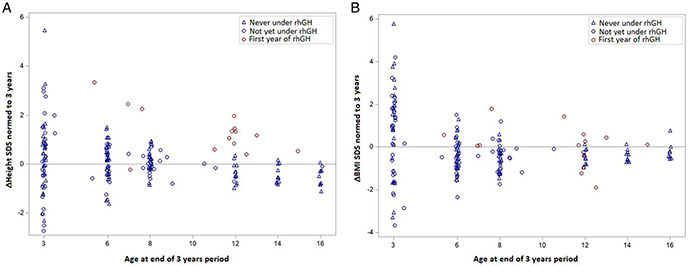
Therapeutic effect of rhGH therapy after 3 years: (A) change in height; (B) change in BMI. Gray line indicates 0 SDS, representing no change in height. rhGH, recombinant human growth hormone; BMI, body mass index; SDS, standard deviation score.

We used a similar model for the analysis of changes in height and BMI SDS over a period of 3 years, using the available values from all 14 participants treated with rhGH for at least 3 years. We found an increase in height SDS change of 1.5 SDS (CI 0.99-2.05, *P* < .001) when compared with controls and patients not yet treated. Figure 3 A shows that the changes in height SDS under rhGH are still greater than those seen in the untreated individuals.

Figure 3B shows the change in BMI SDS, which did not significantly differ between conditions (0.5 SDS, CI 0.0-1.0, *P* = .053).

#### Effect of rhGH on adult height

**Figure 4.**
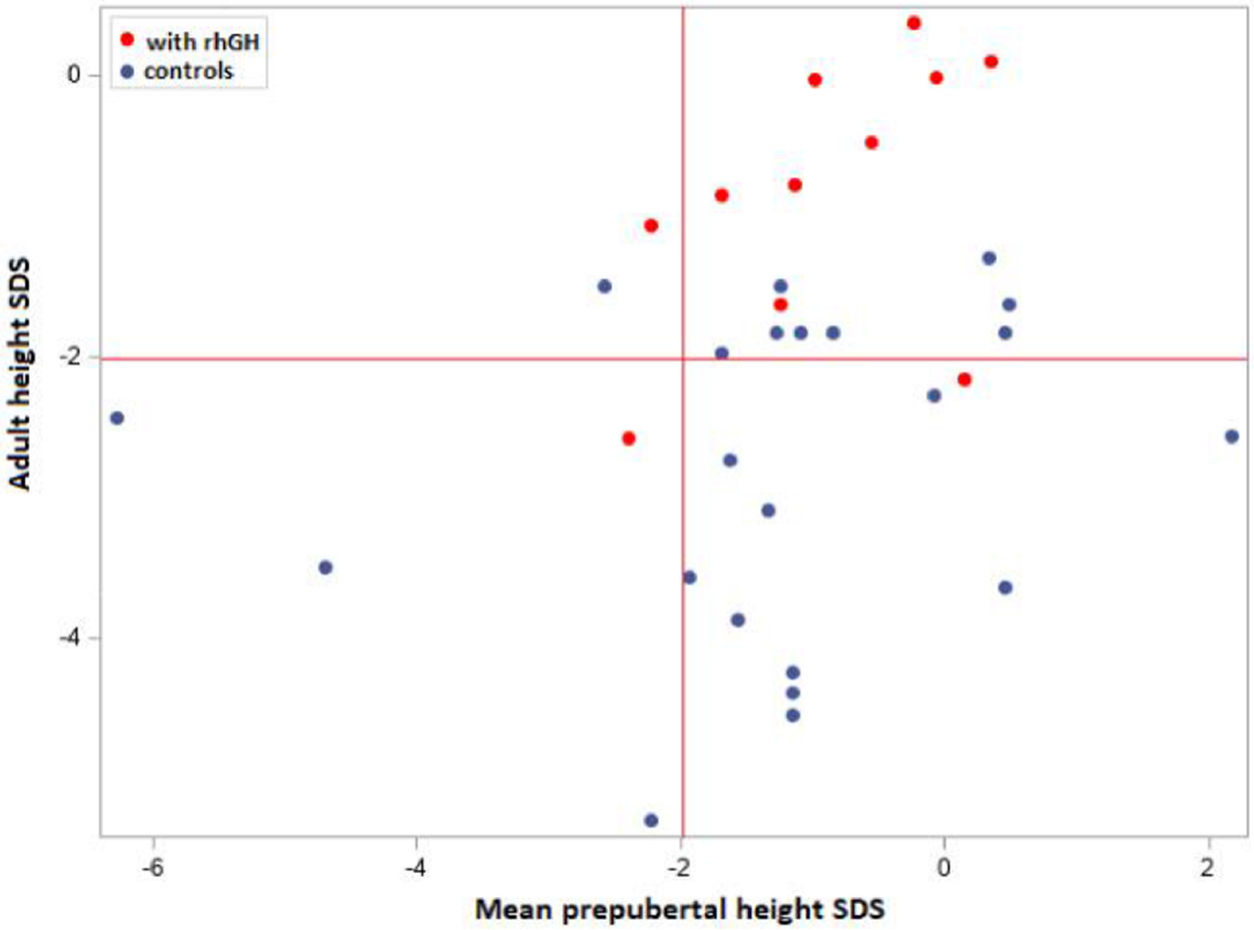
Effect of rhGH on adult height. Red lines indicate the lower limit for height SDS. rhGH, recombinant human growth hormone; SDS, standard deviation score.

To exclude selection bias regarding stature or puberty, we compared the mean height SDS for prepubertal individuals from both groups, over the first 8 years of life (prepubertal development). For this analysis, we compared 11 rhGH-treated participants to 22 controls with available prepubertal measurements. The 2 groups were comparable in regard to height SDS during the prepubertal phase, that is, the median height was −1 SDS (quartiles: −1.7; 0.0) for rhGH-treated individuals and −1.2 (quartiles: −1.7; −0.1) for control individuals, respectively. Figure 4 shows that most of both rhGH-treated and untreated individuals presented a mean prepubertal height between −2 and +2 SDS (right upper and lower quadrants). Interestingly, after adjustment for mean prepubertal height SDS, a significant difference in adult height of 1.9 SDS was evidenced between rhGH-treated and control participants (CI 1.07-2.74, *P* < .001). This difference is shown in Figure 4, where the majority of rhGH-treated participants presented an adult height in the normal range (right upper quadrant), while more than half of the untreated ones fell below −2 SDS.

**Figure 5.**
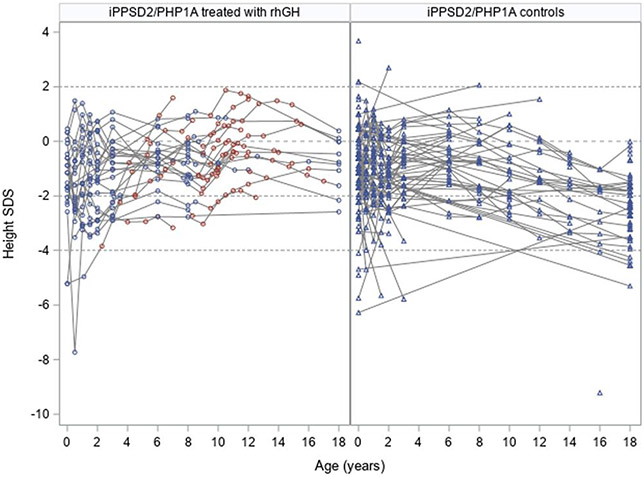
Individual evolution of height in the study group. The iPPSD2/PHP1A participants treated with rhGH are depicted using circles: in blue for the time points before the therapy and in red for the measurements collected under active treatment. The height measurements for the iPPSD2/PHP1A controls are depicted using blue triangles. The gray interrupted lines indicate −2 and +2 SDS, representing the normal range for height.

The individual evolution of height SDS for all iPPSD2/PHP1A participants is shown in Figure 5, using the available measurements.

### Use of concomitant GnRH analogs

Gonadotropin-releasing hormone analogs were additionally used in 10/26 participants. The mean ± SD age at the start of therapy was 10.9 ± 0.5 years; 6/10 were males. In this subgroup, 7 had available adult height measurements, with a range between −1.0 and 0.4 SDS. In the subgroup of participants treated with rhGH, but who did not receive a GnRH analog (*n* = 16), adult height was reached in only 4 patients, ranging from −2.5 to 0.0 SDS.

## Discussion

The lack of data regarding the effect of rhGH on growth and adult height in iPPSD/PHP1A triggered this study. We took advantage of the availability of a large amount of data describing the growth patterns of iPPSD2/PHP1A patients who did not receive rhGH. We used these individuals as age- and gender-matched controls to analyze the effect of rhGH treatment on iPPSD2/PHP1A.

The percentage of participants born SGA in our rhGH-treated iPPSD2/PHP1A group was comparable with that of the controls and also with data from the literature.^14,31^ As expected, in our cohort, the main indication for rhGH was GHD, likely due to GHRH resistance, when considering the already reported high incidence of GHD, of about 50%-60%^19,31,32^; a subgroup of patients was also born SGA and treated with rhGH in the absence of significant postnatal catch-up growth (about 30%^14^).

Recombinant human growth hormone-treated iPPSD2/PHP1A patients were clearly smaller at birth than the untreated study participants (median −1.2 vs −0.9 SDS), but this difference in height vanished progressively during the first years of life. Hence, both groups were comparable in terms of height before entering puberty. We propose that rhGH has a beneficial effect on adult height as the median adult height SDS of iPPSD2/PHP1A controls was below −2 SDS, whereas iPPSD2/PHP1A patients treated with rhGH reached a median adult height of −0.8 SDS. The gain in height occurred mainly during puberty. Mantovani et al.^25^ describe a satisfactory response to rhGH, when the therapy was started in the prepubertal phase and continued throughout puberty, but lack of a pubertal spurt with an adult height below −2 SDS in most of the monitored cases. Even though that study showed that the rhGH response during the first 3 years of rhGH is satisfactory and comparable with the one seen in treated GHD non-iPPSD children, it did lack an iPPSD2 control group of individuals not treated for short stature.^25^ Our results, showing an improvement of both growth and adult height after rhGH therapy, might differ from those of Mantovani et al.^25^ due to the larger sample size of patients analyzed in the first 3 years of therapy and, later, with available adult height measurements, as well as the use of a control population. Recombinant human growth hormone-treated iPPSD2/PHP1A individuals did not decline from their individual percentile during puberty, and they reached a median adult height that was −0.8 SDS below average, with only 1 individual under −2 SDS. We could show, by using an adequate statistical approach, that the difference in adult height is clearly significant between the 2 groups, and that rhGH efficacy is already evident after 1 year of administration. More than half of the iPPSD2/PHP1A individuals not treated with rhGH reached an adult height below −2 SDS, a finding comparable with those of other reports.^14,18^

Some patients were also treated with GnRH analogs for early pubertal development, but our data set is too small to observe a beneficial effect of this treatment when added to rhGH.

In addition, some patients required higher doses of rhGH than 1 might expect when treating GHD; this may be due to additional skeletal dysplasia in the context of PTHrP resistance, with an abnormal IGF-I function in the growth plate. We do know that PTHrP plays a major role in skeletal maturation^20,21^ and that Gsα signaling at the PTHrP-activated PTH1R prevents premature differentiation of proliferating chondrocytes.^33^

Given the role of Gsot in the growth plate, it is not surprising that *GNAS* loss-of-function mutations, leading to an impaired Gsα/cAMP/PKA-signaling pathway, affect the growth process. Moreover, literature data suggest that GH and IGF-I are capable of directly interacting with the PTHrP/PTH1R/Gsα/cAMP/PKA-signaling pathway in bone cell cultures^34-37^ and animal models (eg, model for chronic renal failure and severe secondary hyperparathyroidism).^38^ It is therefore tempting to speculate that rhGH therapy contributes not only to an optimal IGF-I concentration in the growth plate, but also to a decrease, at least in part, in the degree of PTH/PTHrP resistance, by interfering with PTHrP/PTH1R expression and/or function locally.

Thus, the effect of rhGH therapy observed in our cohort could be due to a summative effect on all the factors discussed above. The effect of off-label therapy with rhGH for short stature in iPPSD2/PHP1A without SGA or GHD remains largely unknown. Our own experience regarding this therapy in other PTH/PTHrP resistance disorders, such as iPPSD4 (acrodysostosis type 1), where patients are not GH deficient, suggests a benefit in terms of adult height.^39^

Literature data on BMI show a high risk of early onset obesity in iPPSD2/PHP1A patients, but the variability of the phenotype is rather large.^14^ As expected, we observed a median BMI SDS over 1.5 SDS in both groups during the observation period, even though at birth, the BMI was in the normal range. In iPPSD2/PHP1A patients who did not receive rhGH, we noticed that 71% of the young adults displayed a BMI above 1.5 SDS at the end of the observation period. Some authors found that iPPSD2/PHP1A patients with GHD had a higher BMI, suggesting that the hormonal deficiency may contribute to obesity in patients with *GNAS* loss-of-function mutations.^5,18,19^ We and others^25^ did not see any significant change in BMI during 3 years of rhGH treatment; this suggests that alternative mechanisms are involved in the fat mass development of iPPSD2/PHP1A patients, in accordance with the existing data on overexpression of PTHrP in obesity, in both animals and humans.^40,41^ An interesting hypothesis would be that early obesity is a result of PTHrP resistance in adipose tissue.

The lack of information on GH secretion status in all the controls due to the retrospective nature of the study might be considered a limitation of this study, as we cannot exclude that some of them may suffer from undiagnosed GHD. To overcome this issue, we compared growth patterns during the first 8 years of life in rhGH-treated and untreated cohorts. Before entering puberty, both cohorts of patients had similar height SDS, indicating that this limitation has not caused analysis biases. Additionally, we did encounter a large number of missing BMI values for the rhGH-treated population as well as adult height SDS for the controls. Both points raised above highlight that lack of data is a well-known issue in the field of rare disease research, caused by the absence of specific registries. These obstacles regarding data documentation and availability, especially for the diagnosis and phenotype–genotype correlation of iPPSD, were recently described by Tessaris et al.^42^

The strengths of our study are: (1) data on growth and adult height after rhGH therapy available for the largest cohort analyzed so far, (2) the use of adequate statistical approaches that allowed us to exclude possible confounders, for example, puberty, as responsible for the reported observations, and (3) a well-documented cohort regarding other types of hormone resistance.

## Conclusion

We present data from the first study of the efficacy of rhGH in improving adult height in a large iPPSD2/PHP1A population. Our observations indicate a positive effect of rhGH treatment on short stature and adult height in individuals with this condition. Considering the severe skeletal phenotype present in some iPPSD2 patients, screening for GHD should be routinely performed and started at an early age. Further longitudinal, prospective studies are necessary to identify the factors influencing the growth response of iPPSD2/PHP1A patients to rhGH, including the optimal starting age and duration of rhGH.

## Figures and Tables

**Table 1.** Auxologic characteristics of the study population.

Group	*N*	Min	Max	Lower quartile	Median	Upper quartile
*Birth length (SDS)*						
Controls	119	−6.3	3.7	−1.7	−0.9	0.1
rhGH	23	−5.2	0.5	−2.1	−1.2	−0.5
*Birth BMI (SDS)*						
Controls	98	−5.6	3.5	−0.8	0.1	0.8
rhGH	23	−8.1	1.9	−0.8	0.0	0.8
*Final height SDS*						
Controls	70	−5.3	0.0	−3.0	−2.4	−1.7
rhGH	11	−2.6	0.4	−1.6	−0.8	0.0

These data are only descriptive; therefore, no statistical comparison is provided.

Abbreviations: rhGH, recombinant human growth hormone; BMI, body mass index; SDS, standard deviation score; Min/Max, minimum/maximum; *N*, number of cases.

**Table 2.** Changes in height and BMI SDS in the rhGH-treated group.

Parameter (SDS)	*N*	Mean ± SD	Min	Max
ΔH T1-T0	25	0.6 ± 0.5	−0.3	1.9
ΔH T3-T0	11	1.3 ± 1.0	−0.2	3.4
ΔBMI T1-T0	25	0.0 ± 0.6	−1.1	1.5
ΔBMI T3-T0	11	0.2 ± 1.1	−1.9	1.8

Abbreviations: rhGH, recombinant human growth hormone; BMI, body mass index; SDS, standard deviation score; Min/Max, minimum/maximum; *N*, number of cases; ΔH T1-T0 and ΔBMI T1-T0, height and BMI difference in SDS after 1 year of therapy; ΔH T3-T0 and ΔBMI T3-T0, height and BMI difference in SDS after 3 years of therapy.

## Data Availability

The data that support the findings of this study are available from the corresponding author upon reasonable request.

## Significance

Most patients with pseudohypoparathyroidism 1A (PHP1A) and *GNAS* loss-of-function mutations, i.e.that is, inactivating parathyroid hormone (PTH)/PTHrP -signaling disorder type 2 of maternal inheritance (iPPSD2), present with severe short stature. Phenotypic variability and a lack of data on growth make the decision-making process regarding short stature therapy very difficult, even for experienced pediatric endocrinologists. This is the first study describing longitudinal growth in a large cohort of iPPSD2/PHP1A individuals treated with recombinant human growth hormone, including details on adult height. Our data show that this therapy improves growth and adult height in the analyzed population. We hope that our work will help clinicians in the follow-up and treatment of short stature in iPPSD2/PHP1A and motivate further research in this field.
